# What does the concept of the stem cell niche really mean today?

**DOI:** 10.1186/1741-7007-10-19

**Published:** 2012-03-09

**Authors:** Arthur D Lander, Judith Kimble, Hans Clevers, Elaine Fuchs, Didier Montarras, Margaret Buckingham, Anne L Calof, Andreas Trumpp, Thordur Oskarsson

**Affiliations:** 1 Center for Complex Biological Systems, 2638 Biological Sciences III, University of California Irvine, Irvine, CA 92697-2300, USA; 2 341E Biochemistry Addition, Department of Biochemistry, 433 Babcock Drive, Madison, WI 53706-1544, USA; 3 Hubrecht Institute, Uppsalalaan 8, 3584 CT Utrecht, The Netherlands; 4 Howard Hughes Medical Institute, The Rockefeller University, 1230 York Avenue, New York, NY 10065, USA; 5 Institut Pasteur, CNRS URA2578, Département de Biologie du Développement, 28 rue du Dr Roux, 75015 Paris, France; 6 Dept of Anatomy and Neurobiology, Developmental and Cell Biology, and the Center for Complex Biological Systems, University of California, Irvine, Irvine, CA 92697-1275, USA; 7 Divison of Stem Cells and Cancer, Deutsches Krebsforschungszentrum (DKFZ), Im Neuenheimer Feld 280, D-69120 Heidelberg, Germany; 8 HI-STEM - Heidelberg Institute for Stem Cell Technology and Experimental Medicine, gGmbH, Im Neuenheimer Feld 280, D-69120 Heidelberg, Germany; 9 Heidelberg Institute for Stem Cell Technology and Experimental Medicine (HI-STEM), Im Neuenheimer Feld 280, D-69120 Heidelberg, Germany

## The richness of niche-ness - an introduction

Arthur D Lander

Ideas about stem cells, and how they behave, have been undergoing a lot of change in recent years, thanks to developments in visualizing, monitoring, and manipulating cells and tissues. Curious to find out what impact these changes are having on one of the most enduring and widely accepted metaphors in stem cell biology - the idea of the stem cell niche - *BMC Biology *asked researchers working on a variety of systems to write about their current conception of what a stem cell niche really is.

The answers presented below suggest that the detailed mechanisms underlying niche function are extremely varied. Niches may be composed of cells, or cells together with extracellular structures such as the extracellular matrix (ECM). They may be sources of secreted or cell surface factors - including members of the Notch, Wnt, fibroblast growth factor (FGF), epidermal growth factor (EGF), transforming growth factor (TGF)-β, stem cell factor (SCF), and chemokine families - that control stem cell renewal, maintenance, or survival. They may consist of just a single cell type, or a whole host of interacting cells. They may derive from cells outside the stem cell's lineage, or they may derive primarily from the stem cell's own descendents. In general, there seems to be much more consensus about the fact that stem cells invariably need niches than about the specific mechanisms by which niches do their jobs.

Why should a stem cell need a special environment? This is a pertinent question, given that none of the elementary processes that stem cells rely upon - growing, dividing, differentiating - are unique to stem cells. We can easily imagine three classes of answers:

One possibility is that there are demands placed on stem cells that necessitate special support for viability. For example, the need, imposed by cellular immortality, to minimize the accumulation of genetic damage, may drive stem cells to adopt a peculiar metabolic state that might force them to rely upon other cells nearby for sustenance. This 'nutritive' function of the niche remains a formal possibility, but in most systems few experimental data in support of it have so far emerged.

A second possibility is that niches are agents of feedback control. Recent studies tell us that stem cell pools are not slavishly maintained at a constant size by fixed, asymmetric divisions, but are usually capable of expanding or contracting and, even under homeostatic conditions, may face large stochastic fluctuations. The varied growth factors and cell surface molecules produced by niche cells may share the common goal of controlling stem cell pools. If this is the case, then the niche might best be thought of not simply as an environment conducive to stem cell functioning, but as an apparatus for communicating information about the state of a tissue back to the stem cells that maintain it. An important question to address would then be how niches obtain and relay such information.

A third possibility is that niches are instruments of coordination among tissue compartments. Some of the best evidence for this view comes from work on the hair follicle niche, described below by Elaine Fuchs. There, stem and progenitor cells responsible for maintenance of epidermis, pigmentation, hair, and connective and adipose tissue all interact in close proximity. A need to achieve tight coordination among these different cell populations may be the overriding reason for complex organization of this niche. The possibility that other niches may also be hubs of inter-lineage coordination is certainly an idea worth investigating.

## The *C. elegans *distal tip cell and the concept of a stem cell niche

Judith Kimble

**Figure 1:**
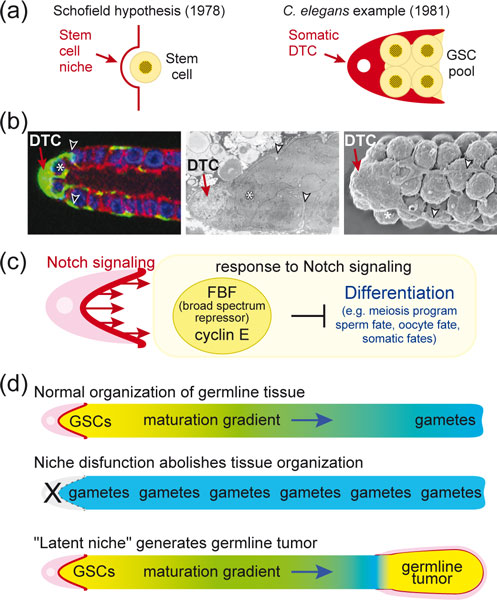
**The *Caenorhabditis elegans *distal tip cell (DTC) and the concept of a stem cell niche**. **(a) **Left, the stem cell niche hypothesis from Schofield [1]; right, the *C. elegans *DTC (red) provides the stem cell niche for the germline stem cell (GSC) pool (yellow). **(b) **Images of the adult DTC and its processes. Left, cytoplasmic green fluorescent protein (green) highlights the DTC and its processes that embrace GSCs. Blue, germline nuclei; red, germline membranes. Modified from [10]. Middle, electron microscopy (EM) image of DTC and its processes. Modified from [10]. Right, scanning EM image of distal gonad; image courtesy of David Greenstein [19]. An asterisk (*) marks one GSC in each image; arrowheads mark processes. **(c) **Molecular view of the niche and its control of GSC self-renewal or differentiation. Dark red, minimalist view of niche as the surface presenting Notch ligands; pink, broader view of niche including DTC as integral to providing the microenvironment for GSC control. **(d) **Expansion of niche concept based on investigations of DTC and Notch signaling in *C. elegans*. See text for explanation.

Schofield originally hypothesized the existence of a microenvironment required for maintenance of stem cells and coined the term stem cell niche [1] (Figure 1a, left). The first example of such a stem cell niche was the mesenchymal 'distal tip cell' (DTC) in *Caenorhabditis elegans *(Figure 1a, right). In this small nematode, a single DTC provides the essential microenvironment, or 'niche', for maintenance of germline stem cells (GSCs; Figure 1b). The DTC is required during development for GSCs to generate the adult germline tissue [2], and in adults to maintain it [3,4]. Both during development and in adults, GSCs are maintained by proximity to the DTC rather than by asymmetric cell division [2-4]. In adults, the DTC extends processes to embrace a pool of GSCs with equivalent potential [3,5], a pool that can regenerate a fully functional germline tissue [6,7]. The simplicity of this niche together with its existence in a genetically tractable organism has made it a paradigm for stem cell control.

The molecular circuitry underlying DTC regulation of GSC maintenance provides the basis for a molecular definition of the niche. Briefly, the DTC uses a signaling pathway that is broadly conserved among metazoans, known as Notch signaling, to regulate GSC maintenance; GSCs respond to Notch signaling via an elaborate network of mRNA and cell cycle regulators (Figure 1c) [8,9]. A major hub of this network is FBF, which is crucial for GSC self-renewal; FBF is a sequence-specific PUF (for Pumilio and FBF) RNA-binding protein and broad-spectrum repressor of differentiation (for example, [10-12]). This FBF hub may reflect the existence of either a fundamental mechanism that acts in many types of stem cells or a specialized mechanism that acts primarily in GSCs to protect their totipotency. A signature of this network is a pervasive redundancy that made the circuitry challenging to unravel experimentally, but renders GSC decisions (self-renewal versus differentiation) highly robust and regulatable [9,10]. So how is the niche defined in molecular terms? A minimalist view is that the DTC membrane presenting Notch ligands to adjacent GSCs defines the niche (Figure 1c, dark red). A broader view includes the DTC itself as integral to the continuous Notch signaling at its surface (Figure 1c, pink).

Investigations of the DTC and Notch signaling have expanded our notion of what a stem cell niche can do. Normally germ cells mature in a gradient, with GSCs at the distal end, differentiated gametes at the proximal end and progressively maturing germ cells in between (Figure 1d). The DTC and Notch signaling establish and maintain that pattern of maturation [2,13], and also regulate formation of normal oocytes at the proximal end of the tissue [14]. Therefore, the influence of the niche extends beyond stem cell control to include the regulation of tissue organization and function.

Investigations of the DTC and Notch signaling also provide insights into the developmental generation of a niche, a process essential for stem cell regulation. The DTC arises from an asymmetric cell division [15], and the Wnt signaling pathway and CEH-22/Nkx2.5 transcription factor specify its niche properties [16,17]. Manipulation of the Wnt pathway and CEH-22 can direct formation of ectopic niches, ectopic GSCs and ectopic maturation gradients [16,17]. In addition, a 'latent niche' was revealed when immature germ cells aberrantly came into contact with non-DTC cells expressing Notch ligands (Figure 1d) [18]. Such a latent niche drives formation of a germline tumor, perhaps because its geometry interferes with the movement of GSC progeny out of the niche.

## The intestinal crypt niche

Hans Clevers

A minimal definition of an adult stem cell involves only two criteria: 1) an adult stem cell persists for the lifetime of the animal ('longevity'); and 2) an adult stem cell can make all cell types of the tissue to which it belongs ('multipotency'). Adult stem cells typically depend on a close interaction with a dedicated cellular environment, the so-called niche. While it has been possible to study invertebrate stem cells and their niches with single-cell resolution, the size of mammalian tissues combined with the infrequent occurrence of stem cells have complicated the identification of individual stem cells *in vivo *[20]. The epithelium of the mammalian small intestine presents a prototypic example of the hierarchical organization of stem cell-driven, self-renewing tissues. A limited number of stem cells reside at the crypt base. Each of these stem cells divides once per day [21]. Daughter cells can exit the stem cell compartment into the transit amplifying (TA) compartment. TA cells undergo approximately four to five rounds of division approximately every 12 hours, an unusually short duration [21]. TA cells differentiate into differentiated cell types, such as enterocytes, goblet cells and enteroendocrine cells, which continue to move up the flanks of the villi. Upon reaching the villus tip after two to three more days, the differentiated cells undergo apoptosis. A fourth cell type, the Paneth cell, also derives from the stem cells, but migrates downwards and settles at the crypt base to live for four to six weeks [22].

**Figure 2:**
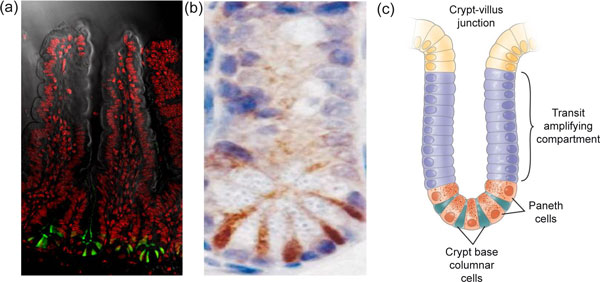
**Expression of Lgr5-GFP at crypt bottoms**. **(a) **Lgrf-5 is shown in green, with a counterstain for DNA in red to outline crypts and villi. **(b) **Lgr5 marks cycling crypt base columnar cells. Lgr5 expression appears in brown, in between the white/blue Paneth cells at crypt bottoms. **(c) **Schematic of crypt architecture. Reproduced, with permission from Elsevier, from Barker N, Clevers H: *Gastroenterology *2007, **133:**1755-1760.

Two competing schools of thought have existed as to the identity of the crypt stem cell before lineage-tracing approaches were developed. Leblond and colleagues originally proposed small cycling cells located between the Paneth cells, the crypt base columnar cells [23,24] as stem cells. Potten proposed the first non-differentiated cell directly above the Paneth cells - the +4 cell - as stem cells. Of note, Potten showed that these cells are not quiescent but cycle every 24 hours [21]. It was recently found that Leblond's crypt base columnar cells specifically express the *Lgr5 *gene, encoding the leucine-rich repeat-containing G-protein coupled receptor 5 [25] (Figure 2). Lineage tracing demonstrated that the Lgr5^hi ^cells generate all cell types of the small intestinal epithelium over the lifetime of the animal, thus fulfilling the above criteria. Similar data were obtained using a *CD133*-based lineage-tracing strategy [26]. As further proof of 'stemness', a single Lgr5^hi ^cell can grow *in vitro *as an ever-expanding epithelial organoid, or mini-gut, that shows all the hallmarks of the *in vivo *epithelial tissue, unveiling an unusual level of architectural self-organization in the absence of a niche consisting of non-epithelial cells [27].

Lgr5 stem cells are closely associated with Paneth cells *in vivo *and *in vitro*. Paneth cells are known to produce bactericidal products, but they also make EGF, TGF-α, Wnt3 and the Notch ligand Dll4, the essential components of the mini-gut culture system [27]. While single sorted stem cells grow inefficiently in culture, stem cell/Paneth cell doublets robustly generate mini-guts. *In vivo*, genetic removal of Paneth cells results in the concomitant loss of Lgr5 stem cells. Thus, Paneth cells, daughters of Lgr5 stem cells, provide essential stem cell niche signals.

**Figure 3:**
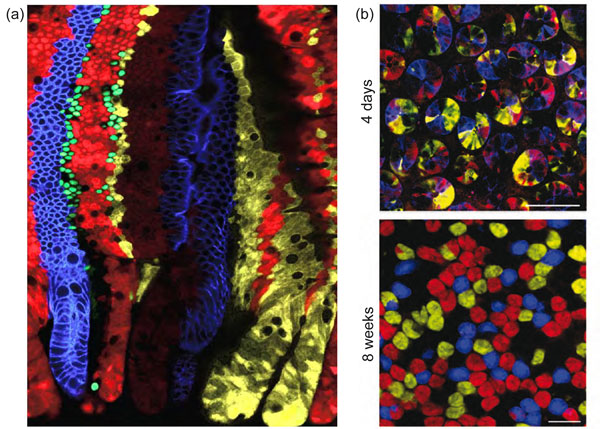
**Stem cells are marked in individual colors by the multicolor Cre reporter Confetti**. **(a) **Each crypt becomes monochromatic over time (bottom of image), producing parallel bands of differently colored cells on villus flanks. **(b) **Confocal sectioning through multiple crypt bottoms. When individual stem cells are marked with different Confetti colors, crypts resolve to monoclonality (that is, they become monochromatic in 4 to 8 weeks due to neutral competition of the stem cells). All images in this figure were reproduced with permission from Elsevier, from Snippert H *et al*.: *Cell *2010, **143:**134-144.

Each crypt contains around 15 stem cells and 15 Paneth cells. As a population, Lgr5 stem cells persist life-long, yet crypts drift towards clonality within a period of one to six months (Figure 3). We have collected short and long-term clonal tracing data of individual Lgr5^hi ^cells. The combined data do not support asymmetric stem cell division. Rather, each crypt appears to provide space for a fixed number of Lgr5^hi ^stem cells. Each day, the resident stem cells double their numbers by symmetric divisions, after which they stochastically adopt stem or TA fates as the outcome of competition for available niche space - the available Paneth cell surface. This determines the number of Lgr5^hi ^stem cells in a crypt. Paneth cell numbers are therefore the key determinant of the stem cell niche and must be tightly regulated under normal homeostatic conditions, which is indeed the case [28]. It will be of interest to understand what determines Paneth cell numbers and their slow turnover rate.

## The hair follicle stem cell niche

Elaine Fuchs

Stem cells reside in specialized microenvironments, known as 'niches' [29]. Cellular components of the niche participate importantly in governing stem cell behaviors, ranging from dormancy and activation to migration and differentiation. Until recently, the niche components impacting on stemness were assumed to derive from heterologous cell types of non-stem cell lineages. Unexpectedly, however, increasing evidence from both invertebrates and vertebrates has begun to broaden this view to include stem cell progeny themselves as important niche components that regulate stem cell activity and behavior.

The skin is the largest organ, and its enormous need for tissue regeneration makes it the most abundant source of stem cells of our body. Hair follicles of the skin are unique in that they undergo synchronized, cyclical bouts of tissue regeneration beginning with a phase in which the hair grows out, followed by a destructive phase in which the hair stops growing and the lower two-thirds of the follicle degenerates. The destructive phase is followed by a period of rest, after which the cycle begins anew. As such, the hair follicle stem cells, which fuel this tissue regeneration, undergo extended periods of rest, and are only briefly activated at the beginning of each hair cycle [30]. Given the beauty of this system, the hair follicle has emerged as an important paradigm to study stem cells in quiescence and in action.

**Figure 4:**
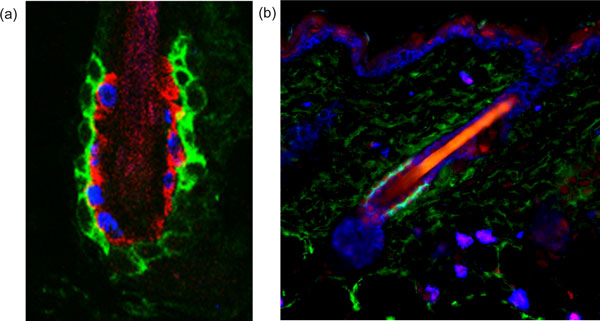
**Homeostasis and repair of the adult tissues depends on tissue-specific stem cells**. **(a) **The architecture of the hair follicle stem cell niche. The hair follicle stem cells are marked by CD34 staining (in green). One of their important niche components is the inner layer of the bulge, marked by K6 staining (in red) and composed of differentiated hair follicle stem cell progeny that underwent the transition from slow-cycling to faster-cycling. This feature was exploited by bromodeoxyuridine (BrdU) nucleotide pulse-chase to mark the inner layer cells with blue BrdU staining in the figure. This inner layer of bulge cells plays a key role in maintaining the quiescence of the outer layer of hair follicle stem cells. This image is courtesy of Y-C Hsu and E Fuchs. **(b) **The hair follicle stem cells are marked by CD34 staining (in green) and are quiescent, due to the high level of bone morphogenetic protein (BMP) signaling within the niche, as shown here by the nuclear staining for phosphorylated Smad1 (in red), the transcriptional effector of the BMP pathway. The nuclei of the skin cells are marked here in Keratin-5 (blue), which reveals the presence of the emerging hair follicle below the activated stem cell niche. This is a classical sign of entry into the growth phase of the new hair cycle. This image is courtesy of N Oshimori and E Fuchs.

Hair follicle stem cells reside in the outermost layer of the 'bulge', an anatomical region located just below the sebaceous glands of the follicles [31,32] (Figure 4a). The bulge niche hosts not only hair follicle stem cells, but also melanocyte stem cells, the latter interspersed between the former [33,34]. The behaviors of these two stem cell populations are well-coordinated, enabling differentiating melanocytes to generate and transfer pigment to the transiently amplifying, committed hair follicle progenitors as they begin to terminally differentiate to produce hair shaft cells. The niche is also surrounded by a basement membrane of ECM, a dermal sheath, and a variety of sensory neurons and blood vessels. Just above the bulge is the arrector pili muscle - responsible for making hairs stand up - which places its mesenchymal stem cells at the crossroads [35]. As such, the bulge niche is a complex but rich milieu of inputs.

An unusual feature of the hair follicle stem cell niche is that one of its key stimulatory components is transient. The dermal papilla is a cluster of specialized mesenchymal cells that rests adjacent to the bulge niche during the resting phase of the hair cycle, but moves downward with the committed proliferative progenitors following transition to the growing phase. During the dormant phase, crosstalk between the dermal papilla and the hair follicle stem cells contributes to the threshold of activating cues (Wnts, bone morphogenetic protein (BMP) inhibitors and TGF-βs) necessary to shift the stem cells from a quiescent to an activated state [36-45].

Another facet of the hair follicle involves the molecular brakes that put its stem cells back into quiescence following an active period of tissue regeneration. In the past year, it was discovered that as hair follicle stem cells progress along their lineages and near completion of the active production of the hair and its channel, some of the terminally differentiated progeny midstream along the lineage wind up back in the bulge. There, they reside in the inner layer that is sandwiched between the outer layer of hair follicle stem cells and the inner core that contains the hair shaft. These invading progeny have lost their potential for stemness and do not regain it even upon wounding. However, they contribute heavily to the niche by transmitting potent BMP and FGF signals that maintain stem cells in a quiescent state [46] (Figure 4b). To reactivate the hair cycle, activating cues must overcome the inhibitory inputs. Compounding these localized niche signals, the balance is also influenced by waves of macro-environmental signals emanating from the dermal adipose tissue [47-49]. These long-range signals help to synchronize the stem cell niches in the hair coat.

Overall, the ease of working with the hair follicle stem cell niche, the abundance of its stem cells, and the synchronized bouts of natural tissue regeneration have catapulted this system to a prominent position in niche research. The complexity of its niche signals and the diversity of stem cells within this niche will keep the field occupied for the decade to come.

## Skeletal muscle: the satellite cell niche

Didier Montarras and Margaret Buckingham

The repair of adult skeletal muscle depends on muscle satellite cells, which, when activated upon injury, will proliferate and then differentiate to make new muscle fibers, or, after self-renewal, re-constitute the reserve of muscle progenitors. The satellite cell therefore displays properties of a tissue-specific stem cell [50]. In normal adult muscle, it is localized as a 'satellite' in close association with the muscle fiber [51], under the basal lamina, which separates individual fibers from the interstitial space. This is the niche of the quiescent satellite cell. There is as yet no clear evidence that the fiber itself regulates the positioning of the satellite cell. Myonuclei lie on the periphery of the contractile apparatus, which occupies the central core of the fiber, although they are spaced along the fiber without obvious synchronization in relation to satellite cells. The fiber is contacted by tendons and nerves and it has been proposed that there is a relationship between myoneural junctions and satellite cell density [52], but this requires further investigation. The interstitial space is mainly occupied by a heterogeneous population of connective tissue cells and blood vessels and there is accumulating evidence that vascularization influences the satellite cell niche [53]. A remarkable feature of skeletal muscle is that the number of satellite cells per fiber does not vary for a given fiber type and is precisely reconstituted after regeneration. Between fiber types this fixed number is different, with a four-fold increase in satellite cells for slow oxidative ('slow twitch') compared to fast glycolytic ('fast twitch') fibers. This phenomenon correlates with the denser network of blood vessels in slow oxidative muscles and more recent investigations have demonstrated that satellite cells are frequently found in the vicinity of blood vessels. There is evidence for crosstalk between satellite cells expressing the receptor Tie2 and neighboring capillary associated cells (for example, pericytes) producing Angiopoietin1, which contributes to the maintenance of quiescence. The Notch pathway has also now been implicated in the maintenance of quiescence. If Notch signaling is disrupted, satellite cells spontaneously activate and differentiate in the absence of injury. Surprisingly, this takes place without proliferation, leading to depletion of satellite cells, so that regeneration is impaired [54,55]. Satellite cells express the Notch receptor, but the source of the ligand required to activate the pathway is not yet clear. However, the muscle fiber is probably the best candidate, since it is in direct contact with the satellite cell and Notch ligands are transmembrane proteins. Furthermore, there is experimental evidence for production of the Notch ligand Delta by the fiber [56].

**Figure 5:**
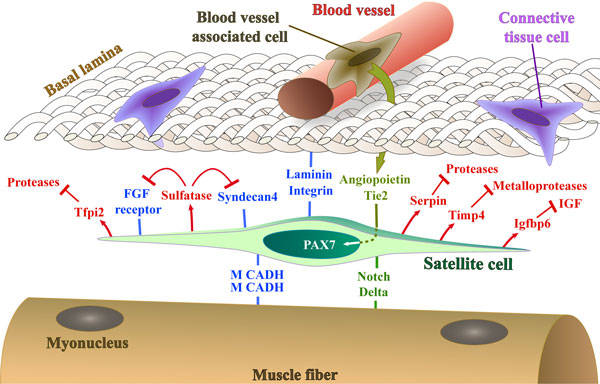
**A representation (not to scale) of the satellite cell, marked by Pax7, in its niche on the muscle fiber under the basal lamina in proximity to a blood vessel**. Cell adhesion molecules, signals received from surrounding tissues, and molecules secreted by the satellite cell that regulate the niche and promote the quiescent state, discussed in the text, are illustrated. IGF, insulin-like growth factor; M CADH, M-cadherin.

The satellite cell is anchored to the surface of the muscle fiber and to the basal lamina, as exemplified by M-cadherin and integrin/laminin interactions, respectively. The notion that the satellite cell actively participates in the building of an environment that maintains quiescence, but allows it to remain poised for activation, is illustrated by *in vivo *expression profiling studies [57]. Quiescent satellite cells are marked by the expression of genes for secreted inhibitors of proteases (Serpin, Tfpi2) and also for tissue inhibitors of metalloproteases, such as Timp4, whereas on activation, when the satellite cell breaks away from its niche, these are rapidly down-regulated, and the satellite cell produces high levels of proteases. Transcripts of proteins associated with cell motility, such as Doublecortin, are also up-regulated on activation. The satellite cell also modulates the activity of signaling molecules, such as FGF, by secreting enzymes involved in de-sulfation that modify proteoglycans in the ECM [58] or growth factor binding proteins such as Igfbp6. In addition to modulating its environment, like other long-lived quiescent stem cells, the satellite cell is also well armed against genotoxic substances and oxidative stress. Thus, the satellite cell of skeletal muscle in its niche on the fiber is subject to signaling from its surroundings (Figure 5) and is also actively involved in maintaining its quiescent state.

## Building one's own nest - what the olfactory epithelium suggests about neuronal stem cell niches

Anne L Calof

Since the central nervous system (CNS) does not regenerate to any significant extent, at least in mammals, it was long assumed that the CNS lacks stem cells (rendering any questions about neuronal stem cell niches moot). In the 1960s, however, investigators such as Joseph Altman and colleagues, using the new technique of injecting ^3^H-thymidine to label cells in S phase, obtained evidence that some CNS glial cells - and a few cells that were apparently neurons (generally defined as being post-mitotic, terminally differentiated cells) - were the progeny of progenitor cells still functioning (dividing) in postnatal rodents [59-61].These progenitor cells were found in the regions near the lateral ventricles of the forebrain (the subventricular zone, or SVZ) and a part of the dentate gyrus of the hippocampus now often referred to, by analogy, as the subgranular zone (SGZ).

Now, five decades later, hundreds of articles have been devoted to the study of neuronal stem cells in these two regions (the SVZ and the SGZ), which still appear to be the only consistent sites of sustained neurogenesis and neuronal regeneration in the mammalian CNS. As a result, a lot is being learned about the nature of the 'niches' that support proliferation, self-renewal, and differentiation of stem cells into neurons and glia in these regions of the brain [62-64]. As one might expect, most signaling molecule families that are important in neural development (EGFs, TGF-ßs, FGFs, Notch, Shh, and others) are also important in the maintenance of stem cells in the adult brain, and can be found in or around these niches [65-67]. It is not surprising, and certainly significant, that regions of the brain that retain characteristics of the embryonic environment in which the brain was generated are crucial for the maintenance of stem cells that retain the capacity for generating neurons. Another very interesting aspect of these CNS niches is that they are invariably juxtaposed to supporting cell tissues: they are found near blood vessels, the ventricles that line the brain (and hence near both ependymal cells and the cerebrospinal fluid these cells produce), or both [68].

Such juxtapostion of neuronal stem cells with non-neural supporting cell tissue is characteristic of a part of the peripheral nervous system that is famous for its ability to maintain lifelong neurogenesis: the olfactory epithelium (OE). The OE generates - and regenerates - olfactory receptor neurons (ORNs) throughout life from stem cells that lie in the basal compartment of the epithelium; and it does so robustly in response to injury (for example, [69] and references therein). Importantly, the OE maintains throughout life striking structural similarities to the neuroepithelial primordia that generate the rest of the nervous system, including its epithelial structure and its dependence on a subjacent stroma derived from mesenchyme and neural crest [70,71]. This stroma is required for the maintenance of stem cell activity, since survival of isolated OE neural stem cells at low density is only possible when they are cultured on stromal feeder cells [72].

**Figure 6:**
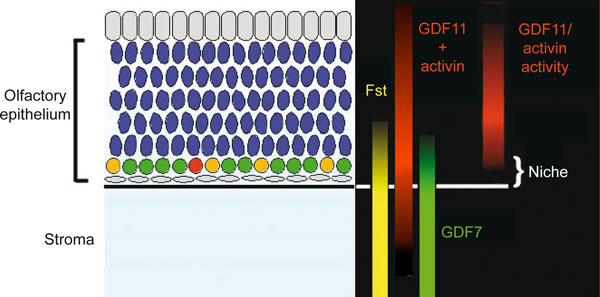
**Model in which molecular gradients along the apical-basal axis of the olfactory epithelium (OE) generate the neuronal stem cell niche**. In the OE, differentiation proceeds in a basal-apical direction, with stem cells (yellow) and intermediate progenitors (shown in red and green) lying in a basal compartment, underneath the post-mitotic olfactory receptor neurons (shown in blue) to which they give rise. Note that localized expression, along with the interaction of growth differentiation factor (GDF)11 and activin with Fst (a high affinity antagonist of both proteins), create a niche within the OE in which the activity of factors that promote neurogenesis (for example, GDF7) is high, and that of factors that inhibit neurogenesis (for example, GDF11, activin) is low.

Given that the OE's neurogenic capacity appears far greater than that of the SVZ or SGZ, the OE presents us with an opportunity to identify basic principles underlying the organization of neuronal stem cell niches. Interestingly, recent studies suggest that the stem cells of the OE play a major role in building their own niche. In particular, the proliferation and self-renewal of OE stem cells appears to be under the control of a host of diffusible factors produced by stem cells, their progeny and their neighbors. For example, OE stem cells and their progeny make morphogens such as GDF11 and activin, which feed back to inhibit stem cell proliferation and self-renewal, providing a mechanism for control of cell number [73-75]. The mesenchymal cells of the stroma, in contrast, make GDF7, which stimulates neurogenesis ([71], and unpublished observations) and follistatin (a secreted molecule that irreversibly inhibits both GDF11 and activin). Given the expected range of diffusion of these sorts of molecules (on the scale of 10 to 100 μm), it quickly becomes apparent that the environment most conducive to stem cell maintenance should exist at the interface between epithelium and stroma (Figure 6), a result that is supported by mathematical modeling [76]. Indeed, as decades of study have shown, the basal compartment of the OE is precisely where its neuronal stem cells reside.

It appears, then, that OE neural stem cells, together with their neighbors, assemble their own niche. The question for the future is whether the same is true for those areas of the CNS that have the capacity to regenerate. Thus, it should be fruitful to take a closer look at SVZ and SGZ development, focusing in particular on how development initially constructs the cellular neighborhood in which the stem cells of these mature structures come to reside.

## Hematopoietic stem cell-niche units in the bone marrow

Andreas Trumpp

Hematopoietic stem cell (HSC) niches in the bone marrow are defined as the cellular and molecular microenvironment that regulates HSC function [77]. This includes control of the balance between dormancy and active self-renewal division as well as progenitor output and early lineage decisions. Niche-derived signals regulate HSC function in conjunction with cell autonomous mechanisms by forming HSC-niche units in which HSCs and niche cells exchange signals to generate a stable, but dynamic and flexible, entity [78]. Most importantly, niches are not only essential for control of HSC function during homeostasis, but niche-derived signals are also critical for the engagement of specific programs in response to stress. Bone marrow stress can be induced by bleeding or by cell loss induced by toxic substances, including chemotherapeutic agents. In addition, bacterial or viral infections and the associated inflammatory responses have a significant effect on HSCs and thus likely also on their niches [79]. However, these issues have only started to be addressed experimentally. The goal of these repair processes in the bone is to rapidly restore homeostasis and have the highly precious HSCs return to a protected dormant state.

It is evident that a prerequisite for studying stem cell niches is detailed knowledge about the identity and precise localization of stem cells themselves. HSCs, which mostly reside in the marrow of the long bones, hips and spine, can be identified and isolated prospectively by multi-parameter flow cytometry (FACS) and show a Lin^neg^Sca1^hi^c-Kit^+^CD34^-^CD48^-^CD150^hi ^phenotype. At the clonal level, they can reconstitute the entire hematopoietic system of lethally irradiated mice and are serially transplantable [80,81]. The population of HSCs as defined above contains at least two subsets. First, active HSCs, which ensure the continuous production of new blood cells during steady-state homeostasis, and second, a numerically smaller HSC population harboring superior self-renewal capacity. During homeostasis this smaller HSC population is retained in a state of dormancy (dormant HSCs). In response to stress, niche signals activate them so that they can be involved in the repair process after injury [81-84]. Both dormant and active HSCs are preferentially found as single stem cells enriched in the trabecular regions of long bones. However, there is significant debate about the more detailed location of HSCs within the marrow, which contains both the endosteal region close to the bone lining osteoblasts (OBs; endosteal niche) and a vascular niche located around small sinusoidal blood vessels associated with various stromal and neuronal elements. While FACS allows us to combine at least eight parameters to identify HSCs *ex vivo*, advanced fluorescence microscope technology used to image HSC-niche units on bone sections is much more limited, making the localization of endogenous HSCs and their niche cells in tissue sections highly challenging [78,85,86].

**Figure 7:**
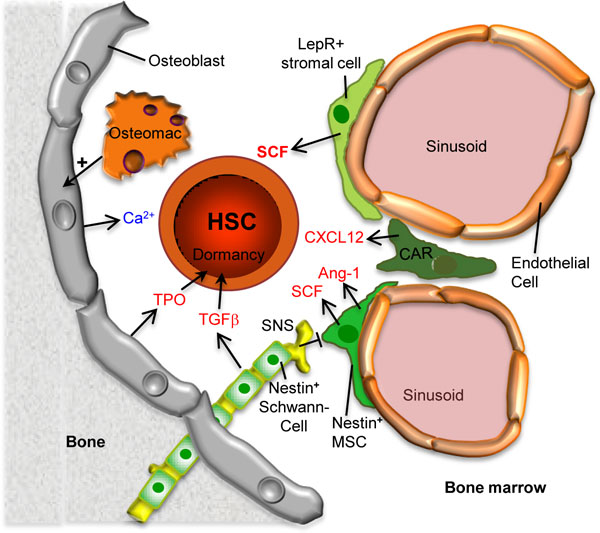
**Model showing the various cell types comprising the bone marrow hematopoietic stem cell (HSC) niche**. The dormant status of HSCs is maintained by transforming growth factor beta (TGF-ß) and thrombopoietin (TPO) produced by nestin+ non-myelinating Schwann cells and osteoblasts, respectively. Stem cell factor (SCF), which is essential for HSC maintenance, is mainly produced by leptin-receptor (LepR)-expressing mesenchymal stromal cells but also by nestin+ mesenchymal stem cells (MSCs) as well as sinusoidal endothelial cells (not shown). The sympathetic nervous system (SNS) negatively affects the activity of nestin+ MSCs. CXCL12 abundant reticular (CAR) cells produce the chemokine CXCL12, which facilitates lodging and engraftment as has been suggested for the high calcium concentration near the endosteal osteoblasts. The four stromal cell populations indicated in green may be somewhat overlapping and the relationship between these cell types remains to be elucidated. Osteomacs are specific macrophages that promote survival to osteoblasts and support nestin+ MSCs (not shown). Ang-1, angiopoietin-1.

Nevertheless, during the past few years a steadily increasing number of cell types have been proposed to regulate HSC function. These include osteoblasts, osteoclasts, macrophages and osteomacs, CXCL12 abundant reticular (CAR) cells, Nestin+ mesenchymal stem cells (MSCs), sympathetic nerves including Nestin+ Schwann cells and finally endothelial cells associated with leptin receptor-expressing stromal cells. Osteoblastic cells were the first cell type identified as a HSC niche component (Figure 7). Recent reports have suggested that specific macrophages named osteomacs combine with OBs to regulate HSC engraftment and granulocyte colony-stimulating factor-induced mobilization [78]. Most recently, additional cellular niche components were revealed by the use of several knock-in reporter mice in which green fluorescent protein was genetically inserted into genes anticipated to be expressed by niche cells. First, HSCs were found to be associated in part with CXCL12-expressing CAR cells [87]. Some of the CAR cells are part of the much smaller population of nestin-expressing stromal cells that contain functional MSCs [88]. The latter express high levels of signaling molecules critical for HSCs, such as CXCL12, vascular cell adhesion molecule 1 (VCAM-1), Ang-1 and SCF. The activity of nestin+ MSCs is regulated, at least in part, by signals derived from macrophages and sympathetic nerves. To make matters even more complex, glial fibrillary acidic protein-expressing non-myelinating Schwann cells of the sympathetic nervous system have also been found within the nestin+ stromal population, although they are clearly distinct from MSCs [89]. Most importantly, these Schwann cells can convert latent TGF-ß into active TGF-ß, which in turn activates the TGF-ß type 2 receptor (RII) expressed by nearby HSCs and which is critical for HSC functionality. The immediate relationship between nestin-expressing MSCs and Schwann cells and what part they contribute to HSC function remain to be addressed.

Finally, expression of SCF, which stimulates the Kit receptor on HSCs, and which is long known to activate a signaling pathway absolutely required for HSC development, maturation and function, has also been studied by knock-in reporter mice [90]. This study suggests that SCF is moderately expressed by endothelial cells of the marrow sinusoids and at higher levels by associated leptin receptor-expressing perivascular stromal (LEPS) cells. Genetic elimination of SCF from both cell types leads to loss of most HSCs, indicating the relevance of these cells for HSC function [90]. Since LEPS cells do not express nestin, they are distinct from MSCs and Schwann cells, but one cannot exclude the possibility that they overlap with CAR cells [91].

In summary, several cell types cooperate to produce secreted and membrane-bound signaling molecules controlling HSC maintenance, fate and function, thus contributing to the formation of the complex HSC-niche unit. These signal/receptor pairs include: SCF/KIT; CXCL12/CXCR4, TGF-ß/TGFß RII, Ang-1/Tie2 and thrombopoietin/MPL and several others with more fine tuning effects on HSCs [77,89-93]. The last three have been suggested to promote dormancy or hibernation, a typical feature of the most potent HSCs during homeostasis [81,94]. Future research will need to decipher the three-dimensional network of the HSC-niche unit, and to dissect the various extracellular signals and how these are translated into HSC fate and function. In addition, it will be important to unravel the architectural, cellular and molecular changes within the HSC-niche units in response to various stress situations, including bacterial and viral infections as well as chemotherapy-induced toxicity. Not only will a better understanding of these processes in mice and humans allow us to understand more clearly the many different facets of HSC biology during homeostasis and stress, but it may also provide direct clinical applications for many disease areas as well as for regenerative medicine.

## Cancer stem cells and metastatic niches

Thordur Oskarsson

During the progression of cancer and formation of metastasis, tumor cells enter the circulation and are seeded to distant organs where they have to resist and overcome a non-permissive environment to survive. These events can occur early and may already have taken place long before diagnosis of the primary tumor [95]. Increasing evidence suggests that, like normal stem cells, tumor-initiating cells, termed cancer stem cells, do not depend solely on cell-intrinsic events but instead rely heavily on the right microenvironment - or niche - to maintain activity and fitness [96]. However, unlike normal stem cell niches, which have evolved for millions of years, resulting in a fine-tuned crosstalk between stem cells and their environment, the cancer - or metastatic - niche evolves in a remarkably short time, resulting in more disordered interactions. The location of metastatic niches is also more loosely defined and can change as the disease progresses. Hypoxic regions, invasive fronts, perivascular sides and normal stem cell niches are all possible locations where metastatic niches can form. Normal stem cell niches are influenced by the stem cells themselves, but the metastatic niche takes this to new heights. Recruitment of inflammatory cells, endothelial cells and myofibroblasts to the metastatic niche leads to a tremendously complex milieu of growth factors, chemokines, hormones, enzymes and ECM that can promote stem/progenitor cell traits [97,98]. The niche that these components form may provide cancer stem cells with the necessary support to survive and grow into overt metastasis.

The qualities of metastatic niches are beginning to be resolved. Despite the somewhat chaotic nature of these niches, interesting parallels can be drawn between them and normal stem cell niches. Certain qualities and molecular interactions within the cancer niche are indeed directly adopted from normal niches. Many of these components are inducers and regulators of stem/progenitor pathways like the Wnt, Notch, Hedgehog, phosphoinositide 3-kinase (PI3K) and JAK-STAT pathways [99,100]. Moreover, evidence is accumulating on the importance of stem cell features in cancer progression and these properties are associated with poor clinical outcome [99]. Intriguingly, evidence supports not only a passive role of the niche maintaining already established stem/progenitor cell traits, but also that niche components can induce the cancer stem cell phenotype in already differentiated cancer cells. In colon carcinoma, myofibroblasts express hepatocyte growth factor (HGF), a ligand of c-Met receptor tyrosine kinase, leading to co-stimulation and enhancement of Wnt signaling in differentiated cancer cells and promoting their stem/progenitor properties [101]. This underscores the importance of the niche and may be a key feature of the cancer niche since the cancer stem cell phenotype may be a rather unstable and context-dependent trait [102-104].

**Figure 8:**
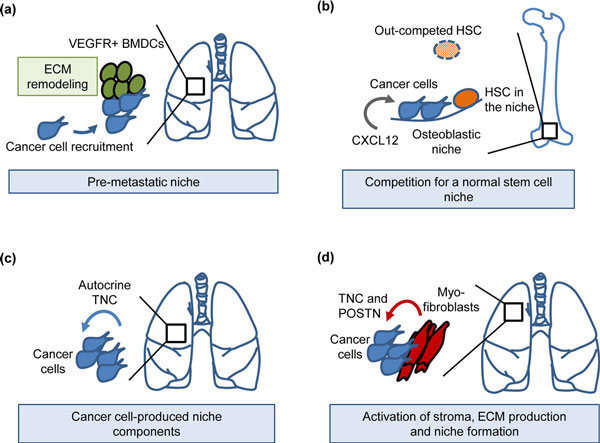
**Examples of metastatic niches during early colonization of distant organs**. **(a) **Systemic changes induced by the primary breast tumor: mobilization of VEGFR1+ bone marrow-derived cells (BMDCs), recruitment to the lungs, extracellular matrix (ECM) remodeling and formation of a pre-metastatic niche [107,108]. The pre-metastatic niche promotes the colonization of breast cancer cells in the lungs. **(b) **Prostate cancer cells enter the osteoblastic niche, competing with hematopoietic stem cells (HSCs) for niche interactions in bone [109]. CXCL12 chemokine promotes colonization of prostate cancer cells in the bone niche via CXCR4 interaction [111]. **(c) **Breast cancer cells bringing their own niche material, tenascin C (TNC), to a distant site thereby promoting early colonization of the lungs [119]. **(d) **Activated myofibroblasts produce the metastatic niche components TNC and periostin (POSTN), resulting in enhanced metastatic outgrowth [119-121]. VEGFR, vascular endothelial growth factor receptor.

The initial events upon entry into distant organs can be critical and most of the cancer cells die soon after extravasation or stay dormant indefinitely [105,106]. Interesting studies have proposed that signaling molecules from the primary tumor may cause changes in distant sites, thereby facilitating metastatic colonization. The environment that this generates has been termed a pre-metastatic niche [107]. Secretion of vascular endothelial growth factor (VEGF)A, placental growth factor (PlGF) and inflammatory cytokines leads to mobilization of VEGF receptor 1 (VEGFR1)-expressing bone marrow-derived cells (BMDCs) and recruitment to the lung where they form a niche that enhances metastatic outgrowth (Figure 8a) [107]. The pre-metastatic niche has also been shown to be enriched for molecules like fibronectin, matrix metalloproteinase 1/2, S100A8/9 and lysyl oxidase (LOX), leading to further recruitment of supportive stromal cells and to ECM remodeling, which together promote the growth of cancer cells entering the niche [108]. To resist the negative forces the cancer cells encounter at distant sites, they take advantage of the molecular interactions and signaling normally active in niches. Interestingly, in some cases cancer cells can even seek out and 'hijack' already established healthy stem cell niches. This has been demonstrated in prostate malignancies, where cancer cells were shown to form micrometastases within HSC niches in the bone marrow and compete with HSCs for the niche interactions (Figure 8b) [109]. The chemokine CXCL12 and C-X-C chemokine receptor 4 (CXCR4), to which it binds, form an axis that is a key molecular interaction between HSCs and the bone niche [110], and is also engaged in bone metastasis of prostate cancer [111]. In addition, other cancers that metastasize to bone also take advantage of this axis. In breast cancer, the CXCL12-CXCR4 axis is enhanced by high Src activity, reinforcing PI3K signaling and promoting survival of cancer cells lodged in the bone [112]. Whether a competition similar to the one seen in the bone marrow niche also occurs in other stem cell niches remains to be seen. However, while the CXCL12-CXCR4 axis is a very important mediator of bone metastasis in cancers like breast-, prostate- and small cell lung cancer, this interaction also mediates metastasis to liver, brain and lungs [113]. Indeed, the chemokine CXCL12 is expressed by myofibroblasts and in hypoxic regions [114,115], both found in various metastatic sites and both potential locations for a metastatic niche.

Important components of the metastatic niche can be expressed by the cancer cells themselves, thereby making cancer cells self-sufficient in this regard since they bring their own niche material to the distant site. The cancer cells that can produce components of a supportive niche on their own will gain a significant advantage upon their arrival in a non-permissive environment. These components can be various growth factors, chemokines or secreted enzymes. Moreover, the ECM can play a significant role in these events. It is increasingly appreciated that the ECM provides more than a structural scaffold for cancer cells and is actively involved in modulating cellular signaling [116]. Indeed, the ECM protein tenascin C (TNC) expressed in normal stem cell niches [117,118] was recently demonstrated to play an important role in metastatic breast cancer. Modulation of stem/progenitor signaling pathways as a result of TNC expression by the cancer cells was shown to be essential to 'jump-start' the growth of lung metastasis in breast cancer (Figure 8c) [119]. The expression of TNC is frequently found in circulating cancer cells isolated from the pleural effusion of patients with systemic breast cancer, suggesting that cancer cell autonomy in TNC production may have a role in the broad and efficient spread of the disease [119]. Moreover, upon activation of the microenvironment, TNC is produced by myofibroblasts and contributes further to metastatic progression [119,120]. In addition to TNC, myofibroblasts produce periostin (POSTN), another ECM protein recently identified as a component of the metastatic niche (Figure 8d) [121]. Interestingly, the role of POSTN in formation of lung metastasis shows a striking similarity to the role of TNC, tempting us to hypothesize that these molecules could be inter-connected or collaborative components of the same supportive system [122]. TNC and POSTN were demonstrated to regulate key signaling pathways involved in the maintenance of cancer stem cell features and activity of Wnt and Notch pathways [119,121]. Disseminated cancer stem cells engage these pathways to resist the inhospitable environment at distant sites.

Today, metastasis is essentially an incurable disease and there is a desperate need for new measures to target metastatic progression. The microenvironment that metastatic cells engage and take advantage of to form a niche is a significant contributor to metastatic outgrowth. Moreover, the niche may possibly also contribute to cancer stem cell resistance to therapeutic intervention. Future studies may lead to identification of niche components that could provide new targets against metastatic progression. Targeting the niche and disrupting the nurturing effect it provides could present us with new means to prevent or even treat metastatic disease.
